# Yogurt drink fortified with menaquinone-7 improves vitamin K status in a healthy population

**DOI:** 10.1017/jns.2015.25

**Published:** 2015-10-16

**Authors:** Marjo H. J. Knapen, Lavienja A. J. L. M. Braam, Kirsten J. Teunissen, Renate M. L. Zwijsen, Elke Theuwissen, Cees Vermeer

**Affiliations:** 1 R&D Group VitaK, Maastricht University, Oxfordlaan 70, 6229 EV Maastricht, The Netherlands; 2 FrieslandCampina, Stationsplein 4, 3818 LE Amersfoort, The Netherlands

**Keywords:** Food fortification, Vitamin K status, *n*-3 PUFA, Vascular health, 25-OH-D, 25-hydroxyvitamin D, cOC, carboxylated osteocalcin, dp-cMGP, desphospho-carboxylated matrix Gla-protein, dp-ucMGP, desphospho-uncarboxylated matrix Gla-protein, MGP, matrix Gla-protein, MK-*n*, menaquinone-*n*, OC, osteocalcin, ucOC, uncarboxylated osteocalcin, VCAM, vascular cell adhesion molecule

## Abstract

Population-based studies have shown an inverse association between dietary menaquinones (MK-*n*, vitamin K_2_) intake, coronary calcification and CHD risk, suggesting a potential role of vitamin K in vascular health. To date, the effects of increased menaquinone intake on (markers of) vascular health have been investigated using predominantly food supplements. Dairy products contain many essential nutrients and can serve as a good matrix for food fortification in order to support health. We were therefore interested to study the effects of a menaquinone-fortified yogurt drink (menaquinone as menaquinone-7 (MK-7); 28 µg MK-7/yogurt drink) on vitamin K status and markers of vascular health. The yogurt drink was also fortified with *n*-3 PUFA, vitamin D, vitamin C, Ca and Mg to support vascular and/or general health. Healthy men (*n* 32) and postmenopausal women (*n* 28) with a mean age of 56 (sd 5) years received either basic or fortified yogurt drink twice per d for 12 weeks. MK-7 was efficiently absorbed from the fortified yogurt drink. Levels of circulating MK-7 were significantly increased from 0·28 to 1·94 ng/ml. In accordance, intake of the fortified yogurt drink improved vitamin K status, as measured by significant decreases in uncarboxylated osteocalcin and desphospho-uncarboxylated matrix Gla-protein. No effects were, however, seen on markers of inflammation, endothelial dysfunction and lipid metabolism. In summary, consumption of a yogurt drink fortified with low doses of among others MK-7 for 3 months significantly improved vitamin K status in a healthy population.

Several population-based studies have shown an inverse association between dietary menaquinone-*n* (MK-*n*; vitamin K_2_) intake and CHD risk, suggesting a potential role of vitamin K in vascular health^(^^1^^–^^3^^)^. Vitamin K serves as a cofactor for γ-glutamate carboxylase, promoting the post-translational conversion of glutamate residues into γ-carboxyglutamate (Gla) in so-called Gla-proteins^(^^4^^–^^6^^)^. The Gla-residues confer Ca-binding properties needed for the proper functioning of these proteins. Of all known Gla-proteins, osteocalcin (OC, synthesised by osteoblasts) and matrix Gla-protein (MGP, synthesised primarily by vascular smooth muscle cells) are investigated most extensively. During vitamin K insufficiency, carboxylation proceeds to a lesser extent, resulting in the release of Gla-proteins in the circulation as undercarboxylated species. Circulating uncarboxylated OC (ucOC) and desphospho-uncarboxylated MGP (dp-ucMGP) are recognised markers for bone and vascular vitamin K status, respectively. Remarkably, substantial fractions of OC and MGP circulate as uncarboxylated species in most healthy adults^(^^7^^–^^10^^)^, suggesting that vitamin K insufficiency is widespread in Western society. High levels of ucOC form an independent risk predictor for bone fracture and low bone mineral density^(^^11^^–^^16^^)^. High levels of dp-ucMGP were found especially in subjects at increased risk for CVD^(^^17^^,^^18^^)^ and have been associated with arterial calcification and cardiovascular mortality^(^^19^^,^^20^^)^. OC and MGP carboxylation can be improved by increased vitamin K intake by diet^(^^21^^,^^22^^)^ and supplements^(^^7^^,^^8^^,^^23^^)^.

Dietary forms of vitamin K are phylloquinone (vitamin K_1_) and the group of menaquinones. Food sources of phylloquinone are green vegetables and several plant oils^(^^24^^–^^26^^)^, whereas menaquinones are primarily found in meat and egg yolk (short-chain MK-4) and in fermented foods, like cheese and curd (MK-7 to MK-10, also referred to as long-chain menaquinones)^(^^26^^)^. Whereas all forms of vitamin K appear to be initially associated with TAG-rich lipoproteins, the long-chain menaquinones are also associated with LDL. These data suggest that the menaquinones have different transport pathways and distribution, and they are therefore thought to be the most adequate form of vitamin K to supply extra-hepatic tissues, such as bone and the arterial wall.

In earlier studies on the beneficial effects of increased vitamin K intake, supplements (tablets, capsules) were used almost invariably. Dairy products contain many essential nutrients with recognised health benefits^(^^27^^)^ and can serve as an ideal matrix for food fortification in order to support health. For this study, we fortified a yogurt drink with menaquinones (in the form of MK-7) and *n*-3 PUFA (EPA + DHA^(^^28^^–^^30^^)^) to support heart health. Vitamin D, vitamin C, Ca and Mg were also added to support general health. First, we studied absorption of vitamins from the fortified yogurt drink. Next, we investigated effects of the fortified yogurt drink on markers of vitamin K status, inflammation, endothelial dysfunction and lipid metabolism. We hypothesised that the fortified yogurt has beneficial effects on vitamin K status and markers of cardiovascular health.

## Experimental methods

### Study design

Healthy men and postmenopausal women between 45 and 65 years of age were recruited from the southern region of Limburg, the Netherlands, through advertisements in local newspapers. Exclusion criteria were: <2 years postmenopausal, BMI <20 and >30 kg/m^2^, hypertension, hypercholesterolaemia, metabolic or gastrointestinal diseases, chronic degenerative or inflammatory diseases, diabetes mellitus, coagulation disorders, cows’ milk allergy and/or lactose intolerance, abuse of drugs and/or alcohol, use of corticosteroids, oral anticoagulants, blood pressure-lowering medication, and/or cholesterol-lowering medication, use of vitamin K supplements, and high dietary intake of vitamin K (assessed by interviews and questionnaires). If subjects were eligible for inclusion, blood was taken at a screening visit in order to select participants with normal or low vascular vitamin K status (dp-ucMGP values >150 pmol/l). After a final health check (interviews and questionnaires), sixty participants (thirty-two men and twenty-eight women) were included in the study and randomly assigned (computer-generated random permutation procedure with stratification for sex) to receive either a basic yogurt drink (control group) or a fortified yogurt drink twice per d for 12 weeks. Both the participants and the study personnel were blinded as to the composition of the drinks (double-blind design). In the period starting at 2 weeks before the start of the study until the final blood sampling, participants were asked to restrict their intake of vitamin K-rich foods. Participants visited the research site at baseline and after 4, 8 and 12 weeks for blood sampling, and for measurements of body weight and height (at baseline). They were instructed to report any signs of illness, medication used and any deviations from the study protocol. In addition, subjects were urged not to change their level of physical exercise or alcohol consumption during the study.

### Ethics statement

This study was conducted according to the guidelines laid down in the Declaration of Helsinki and all procedures involving human subjects were approved by the Medical Ethics Committee of the Maastricht University Medical Centre (Maastricht, The Netherlands). Written informed consent was obtained from all subjects. Trial registration code: clinicaltrials.gov: NCT01672099.

### Study products

The yogurt drinks (250 ml/container) were provided in two batches by FrieslandCampina. The yogurts were coded and subsequently delivered directly to VitaK (Maastricht, The Netherlands) as complete ready-to-use end-products. During visits at the research site or during home visits, study products were handed out to the study participants every 2 weeks. Leftovers had to be returned to assess compliance. The basic yogurt drink was manufactured according to standard procedures (Table 1). The fortified yogurt drink was enriched with MK-7 and *n*-3 PUFA to support vascular health (Table 1). In addition, various basic nutrients were added to support general health: vitamin D, vitamin C, Ca and Mg. The chosen amounts represent 15 % of the recommended dietary intake. The participants consumed two 250 ml yogurt drinks daily; one drink during breakfast and one during dinner. We chose a daily dose of MK-7 (56 µg) that is consistent with the lower level in the commercially available MK-7 supplements, i.e. 45–200 µg, and suggested to beneficially affect bone and heart health. A study duration of 12 weeks was chosen based on earlier findings showing significant effects on OC and MGP carboxylation with nutritional doses of MK-7^(^^7^^)^. We used pure, natural, minimally processed, non-encapsulated marine oil rich in long-chain *n*-3 PUFA as EPA + DHA. The active biological compound MK-7 (MenaQ7) was supplied by NattoPharma. To verify the stability of MK-7, three samples of the study products were analysed at the start and end of the intervention period. The MK-7 content of the fortified yogurt drinks was stable over time.

**Table 1.** Study product composition

Per 100 ml	Standard drinking yogurt	Fortified drinking yogurt
DM (%)	13·7	14·4
Protein (%)	2·6	2·5
Milk fat (%)	1·0	1·1
Saccharides (%)	5·3	5·3
Vitamin K_2_ (MenaQ7) (μg)	Below detection limit	11·65*
Fish oil (EPA + DHA) (mg)	<2	36·3*
Vitamin D_3_ (IU)	<0·1	0·37*
Ascorbic acid (mg)	0·6	23·5*
Ca (mg)	77	108*
Mg (mg)	7·5	58*

* 15 % of the recommended dietary intake.

### Blood sampling

Fasting venous blood was collected by venepuncture for the preparation of serum and plasma (Vacutainer; Greiner Bio-One BV). All blood samples were drawn between 08·00 and 11·00 hours by experienced research nurses. For plasma preparation, blood (10 ml) was collected in citrate tubes, centrifuged at 3000 ***g*** for 15 min, and plasma was divided into aliquots and stored at −80°C until analysis. For serum preparation, blood (10 ml) was allowed to clot for 30 min at room temperature, centrifuged, and the serum was stored at −80°C.

### Circulating markers

Circulating MK-7 levels were measured at baseline and after 12 weeks with a standard HPLC technique using a C18 reversed-phase column and fluorometric detection after post-column electrochemical reduction^(^^26^^)^. Serum ucOC and carboxylated OC (cOC) concentrations were determined with commercial dual-antibody ELISA tests (Takara Shuzo Co. Ltd). Plasma dp-ucMGP and desphospho-carboxylated MGP (dp-cMGP) levels were measured by in-house dual-antibody ELISA tests^(^^17^^)^. An in-house control plasma pool was run on all ELISA plates. To minimise inter-assay variation, the different time-point samples of each subject were analysed on the same ELISA plate, in ascending order of randomisation. In addition to measurements at baseline and at 12 weeks, these markers for vitamin K status were also measured after 4 and 8 weeks of the 12-week intervention period in order to determine more precisely when a significant effect was achieved.

Markers of inflammation, endothelial dysfunction and lipid metabolism were measured with commercial immunoassays at baseline and at the end of the intervention. The following markers were measured: vascular cell adhesion molecule (VCAM), intercellular adhesion molecule and E-selectin (R&D Systems Europe); serum amyloid A, vascular endothelial growth factor, IL-6 and TNF-α (Invitrogen); von Willebrand factor (Abnova); secretory type-II phospholipase A-2 (Wuhan USCN Business Co.). C-reactive protein (inflammation marker), total cholesterol and TAG (markers of lipid metabolism) were measured in serum using fully enzymic techniques on a clinical chemistry analyser. HDL-cholesterol was measured with the use of a homogeneous colorimetric technique. LDL-cholesterol was calculated by the Friedewald formula^(^^31^^)^. Circulating vitamin D (25-hydroxyvitamin D; 25-OH-D) was measured by the automated IDS-iSYS method (IDS plc). Vitamin C (ascorbic acid) levels were measured by a commercial kit (Abcam).

All samples were measured in duplicate. Within- and between-assay precisions were <5 and <10 %, respectively, for all analytical procedures.

### Statistics

**Fig. 1.**
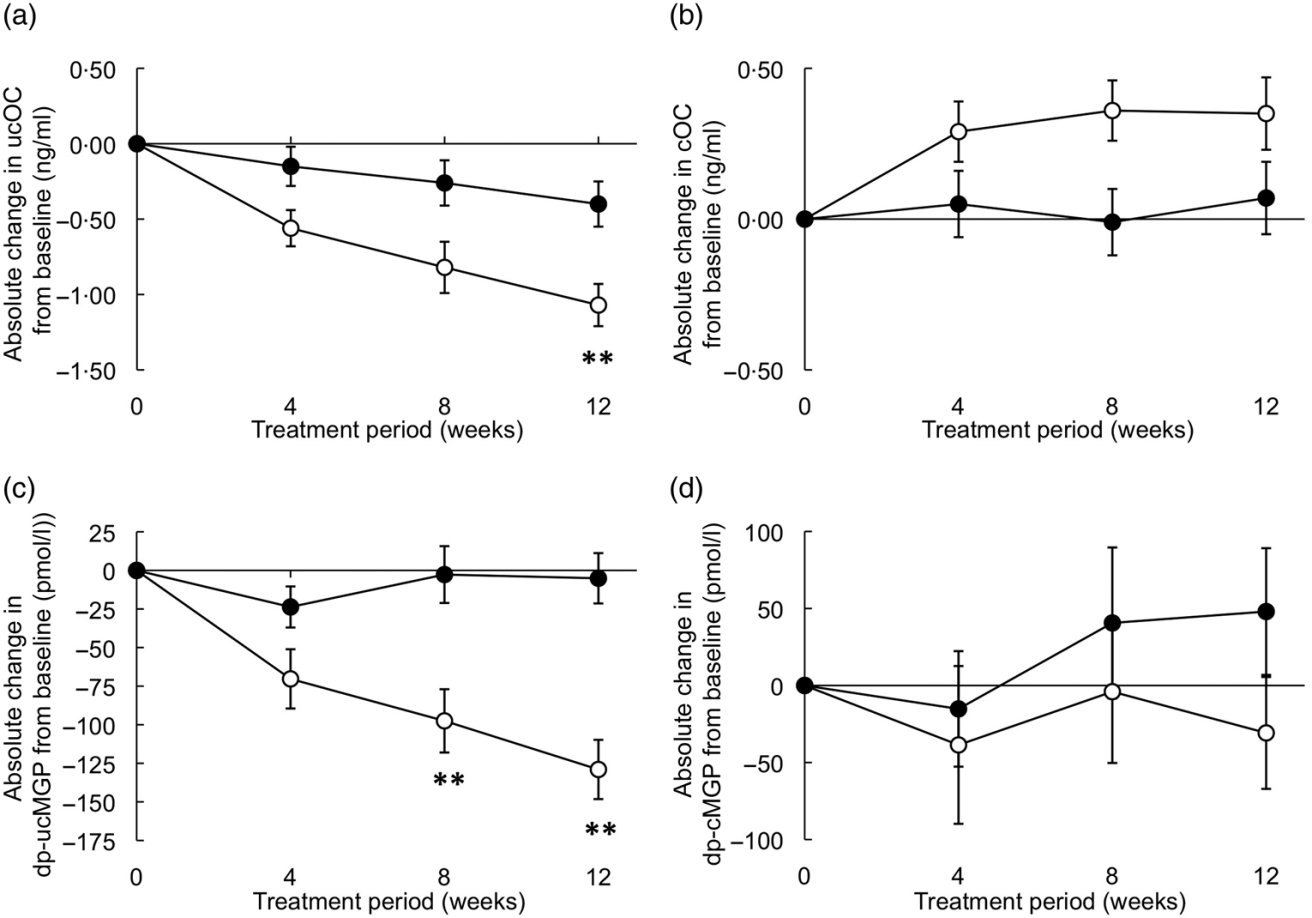
Effects of 12-week consumption of a fortified yogurt drink on markers of vitamin K status. Participants received twice per d either a basic yogurt drink (control group; ●) or a fortified yogurt drink (fortified with menaquinone-7, EPA + DHA, vitamin D, vitamin C, calcium and magnesium; ○) for 12 weeks. (a) Uncarboxylated osteocalcin (ucOC); (b) carboxylated osteocalcin (cOC); (c) desphospho-uncarboxylated matrix Gla-protein (dp-ucMGP); (d) desphospho-carboxylated matrix Gla-protein (dp-cMGP). Values are means (*n* 56), with standard errors represented by vertical bars. ** Mean value was significantly different from that for the control group (*P* < 0·017; correction for multiple comparisons, one-way ANOVA).

The primary outcome measure was circulating dp-ucMGP (vascular vitamin K status). Based on preliminary estimates of the standard deviation in ucMGP in healthy subjects, we determined that thirty participants were required in each group to have a statistical power of 90 % to detect a 15 % difference between treatment groups while allowing for a withdrawal of 10 %. The distribution of variables was analysed by normality tests, boxplots and histograms. Serum amyloid A, IL-6, secretory type-II phospholipase A2 and C-reactive protein were log-transformed. Other variables are presented as means with standard deviations or standard errors (Fig. 1). The baseline characteristics of both treatment groups were compared with a Fisher's exact test for categorical variables (sex and smoking) and an independent-samples *t* test for continuous variables. Statistical significance is reached if *P* < 0·0024 (Bonferroni correction for multiple comparisons, i.e. twenty-one different circulating markers). Correlation analyses at baseline were performed using the Pearson test (two-sided significance level *P* < 0·05). The repeated-measurements ANOVA was used to test between-group and within-group differences for changes of markers of vitamin K status (dp-ucMGP and ucOC) at the different time points. The *P* value was adapted for multiple comparisons between three time points (i.e. absolute changes after 4, 8 and 12 weeks), meaning that *P* < 0·017 was considered significant. Multivariate linear regression analysis was performed to test for associations between treatment and the markers of interest, which were treated as dependent variables (i.e. end values after 12 weeks of circulating vitamins, and markers of vitamin K status, inflammation, endothelial dysfunction and lipid metabolism). The corresponding baseline value and the treatment regimen (standard/fortified yogurt) were included as independent variables and age, BMI and TAG were included as potential confounders. Analysis of the markers of lipid metabolism was performed with age and BMI as confounder. *P* < 0·0024 was considered significant (Bonferroni correction). The potential confounders sex and smoking were not included in the analyses, because they were equally divided over the two treatment groups (see Table 2) and did not contribute to the results. Statistics were performed using SPSS for Windows, version 19 (SPSS Inc.).

**Table 2.** Baseline characteristics of the total study group and of the groups consuming the standard yogurt or the fortified yogurt (Mean values and standard deviations; numbers of subjects and percentages)

	Total group (*n* 56)		Standard yogurt (*n* 29)		Fortified yogurt (*n* 27)		P*
	Mean	sd	Mean	sd	Mean	sd	
Sex (*n*)							0·49
Male	30		15		15		
Female	26		14		12		
Age (years)	56	5	56	5	56	5	0·76
Weight (kg)	75	13	73	13	79	12	0·08
BMI (kg/m^2^)	26	3	25	3	26	2	0·33
Smoking							0·59
*n*	10		5		5		
%	18		17		19		
Vitamins							
MK-7 (ng/ml)	0·25	0·24	0·21	0·21	0·28	0·28	0·29
Vitamin C (ng/ml)	41·4	13·2	43·4	14·6	39·2	11·4	0·22
25-OH-D (ng/ml)	37·6	10·8	39·9	7·6	35·0	13·1	0·09
Vitamin K status							
ucOC (ng/ml)	3·51	1·75	3·47	1·60	3·57	1·93	0·83
cOC (ng/ml)	5·00	1·31	5·04	1·29	4·96	1·34	0·81
dp-ucMGP (pmol/l)	442	187	405	157	481	211	0·13
dp-cMGP (pmol/l)	1812	425	1781	342	1847	508	0·57
Markers of inflammation							
IL-6 (pg/ml)†	0·77	0·63	0·67	0·35	0·88	0·82	0·62
sPLA-2 (pg/ml)†	3681	4137	3594	3044	3776	5124	0·30
vWF (pg/ml)	1007	329	962	288	1056	367	0·29
TNF-α (pg/ml)	1·24	0·67	1·10	0·49	1·54	0·74	0·022
CRP (mg/l)†	1·50	1·44	1·63	1·66	1·35	1·16	0·50
Markers of endothelial dysfunction							
VEGF (pg/ml)	238	131	244	154	233	104	0·76
VCAM (ng/ml)	728	347	647	213	816	436	0·07
ICAM (ng/ml)	216	41·6	207	45	225	37	0·12
SAA (μg/ml)†	18	16	19	19	16	13	0·88
E-selectin (pg/ml)	29	13	30	14	29	11	0·97
Markers of lipid metabolism							
tCHOL (mmol/l)	5·89	1·03	5·91	1·06	5·87	1·02	0·88
LDL (mmol/l)	3·84	0·95	3·82	0·95	3·85	0·97	0·91
HDL (mmol/l)	1·39	0·43	1·42	0·45	1·36	0·42	0·62
TAG (mmol/l)	1·48	0·67	1·43	0·61	1·47	0·67	0·87

MK-7, menaquinone-7; 25-OH-D, 25-hydroxyvitamin D; ucOC, uncarboxylated osteocalcin; cOC, carboxylated osteocalcin; dp-ucMGP, desphospho-uncarboxylated matrix Gla-protein; dp-cMGP, desphospho-carboxylated matrix Gla-protein; sPLA-2, secretory type-II phospholipase A2; vWF, von Willebrand factor; CRP, C-reactive protein; VEGF, vascular endothelial growth factor; VCAM, vascular cell adhesion molecule; ICAM, intercellular adhesion molecule; SAA, serum amyloid A; tCHOL, total cholesterol.

* *P* values of the between-group analysis of both treatment groups at baseline (Fisher's exact test for categorical data (sex and smoking) and the independent-samples *t* tests for continuous data). No significant differences were found (multiple comparisons: *P* < 0·0024).

† Analysis between the subjects consuming the standard yogurt and consuming the fortified yogurt was performed after logarithmic transformation of the values.

## Results

### Baseline characteristics

Baseline characteristics are shown in Table 2. Of the sixty participants, four dropped out (two men and two women) during the study: three participants (*n* 2 in fortified yogurt group, *n* 1 in the control group) disliked the study product and one participant (*n* 1 in fortified yogurt group) was no longer eligible for inclusion because of starting oral anticoagulant treatment. The results are therefore given for the fifty-six participants who completed the study. Because no between-group differences were found between men and women, except for TAG which was higher in men than in women (1·73 (sd 0·70) mmol/l and 1·19 (sd 0·51) mmol/l, respectively; *P* = 0·002), data of men and women were combined in both treatment groups. No significant differences were found between the treatment groups.

At baseline, dp-ucMGP and dp-cMGP correlated significantly with the inflammation marker IL-6 (*r* 0·321 and *r* 0·308, respectively; *P* < 0·05) and with markers of endothelial dysfunction von Willebrand factor (*r* 0·395 and *r* 0·393; *P* < 0·01) and VCAM (*r* 0·399 and *r* 0·653; *P* < 0·001). Circulating ucOC and cOC did not correlate with markers of vascular health.

### Absorption of vitamins

Increased levels of circulating MK-7 (as compared with baseline) were observed after consumption of the fortified yogurt drink during 12 weeks (mean difference: 1·66 (sd 0·96) ng/ml). This increase differed significantly from the change in the control group (mean difference: −0·01 (sd 0·22) ng/ml; *P* < 0·0001). After adjustment for baseline MK-7 values, age, BMI and TAG, the end difference in plasma MK-7 concentrations between the treatment groups remained significant (Table 3). The water-soluble vitamin C was well absorbed, which can be seen from the unchanged levels (from 43·5 (sd 14·4) to 43·6 (sd 18·2) ng/ml; *P* = 0·952) in the control group compared with the significant increase (from 39·1 (sd 11·7) to 46·1 (sd 13·0) ng/ml; *P* = 0·002) in the group using the fortified yogurt drink. For the fat-soluble vitamin D the data are compromised by the fact that – probably due to seasonal effects – in the control group circulating 25-OH-D decreased significantly from 39·9 (sd 7·6) to 31·1 (sd 6·2) ng/ml (*P* < 0·0001); the concomitant decrease in the fortified group (from 35·0 (sd 13·1) to 32·5 (sd 11·2) ng/ml; *P* = 0·081) was much lower, but whether this was the result of vitamin D absorption from the yogurt drink cannot be concluded from the present data. The end values of circulating vitamin C and 25-OH-D between both treatment groups showed no significant differences (independent-samples *t* test: *P* = 0·57 and *P* = 0·55, respectively). However, regression analysis, adjusted for age, BMI and TAG, resulted in a significant increase of vitamin C (*P* = 0·048) and 25-OH-D (*P* = 0·005) levels after 12 weeks’ consumption of fortified yogurt compared with consumption of the standard yogurt (Table 3). After correction for multiple comparisons the significance was lost for vitamin C, but the difference between both yogurts in 25-OH-D was still borderline significant.

**Table 3.** Effect of 12-week consumption of fortified yogurt compared with standard yogurt on markers for vitamin K status, inflammation, endothelial dysfunction and lipid metabolism* (β-Coefficients and 95 % confidence intervals)

	β	95 % CI	*P*†
Vitamins			
MK-7 (ng/ml)	0·85	0·29, 1·40	0·004
Vitamin C (ng/ml)	5·79	0·04, 11·54	0·048
25-OH-D (ng/ml)	3·75	1·17, 6·33	0·005
Vitamin K status			
ucOC (ng/ml)	−0·70	−1·10, −0·29	0·001
cOC (ng/ml)	0·20	−0·16, 0·57	0·27
dp-ucMGP (pmol/l)	−96	−146, −46	<0·0001
dp-cMGP (pmol/l)	−66	−174, 42	0·23
Markers of inflammation			
IL-6 (pg/ml)‡	−0·08	−0·25, 0·08	0·30
sPLA-2 (pg/ml)‡	−0·03	−0·15, 0·09	0·63
vWF (pg/ml)	66	−31, 162	0·18
TNF-α (pg/ml)	−0·15	−0·51, 0·20	0·38
CRP (mg/l)‡	0·13	−0·02, 0·29	0·10
Markers of endothelial dysfunction			
VEGF (pg/ml)	−0·8	−20·2, 18·6	0·93
VCAM (ng/ml)	1·4	−70·9, 73·6	0·97
ICAM (ng/ml)	5·6	−6·6, 17·7	0·36
SAA (μg/ml)‡	−0·02	−0·17, 0·12	0·76
E-selectin (pg/ml)	−0·71	−4·23, 2·81	0·69
Markers of lipid metabolism			
tCHOL (mmol/l)	−0·08	−0·45, 0·29	0·68
LDL (mmol/l)	−0·09	−0·41, 0·24	0·61
HDL (mmol/l)	−0·07	−0·17, 0·03	0·18
TAG (mmol/l)	0·11	−0·07, 0·29	0·23

MK-7, menaquinone-7; 25-OH-D, 25-hydroxyvitamin D; ucOC, uncarboxylated osteocalcin; cOC, carboxylated osteocalcin; dp-ucMGP, desphospho-uncarboxylated matrix Gla-protein; dp-cMGP, desphospho-carboxylated matrix Gla-protein; sPLA-2, secretory type-II phospholipase A2; vWF, von Willebrand factor; CRP, C-reactive protein; VEGF, vascular endothelial growth factor; VCAM, vascular cell adhesion molecule; ICAM, intercellular adhesion molecule; SAA, serum amyloid A; tCHOL, total cholesterol.

* The effect of the fortified yogurt on the dependent variable (end-value of the markers) was adjusted for the baseline values of the concomitant variable, age, BMI and TAG. The effects on markers of lipid metabolism were adjusted for age and BMI.

† *P* values of the multivariate regression analysis were regarded statistically significant if *P* < 0·0024 (Bonferroni correction for multiple comparisons).

‡ Analysis between the subjects consuming the standard yogurt and consuming the fortified yogurt was performed after logarithmic transformation of the values.

### Markers of vitamin K status

Both ucOC and dp-ucMGP decreased during the 12 weeks’ consumption of the fortified yogurt, although only the decrease in dp-ucMGP was significant (repeated-measures, within-subject analysis: *P* = 0·011). Significant between-group differences were found for circulating dp-ucMGP already after 8 weeks (*P* = 0·008), but 12 weeks of intake were needed to significantly decrease ucOC levels (*P* = 0·001).

Circulating ucOC decreased by 33 (sd 18) % after consuming the fortified yogurt for 12 weeks, compared with −7 (sd 26) % in the group drinking the standard yogurt (*P* < 0·0001). Circulating dp-ucMGP decreased by 24 (sd 13) % in the group consuming the fortified yogurt drink, whereas dp-ucMGP levels remained unchanged in the group with the standard yogurt (−1 (sd 28) %; *P* < 0·0001, see Fig. 1(a), 1(c)). Changes in circulating cOC and dp-cMGP levels did not differ between the two treatment groups (Fig. 1(b), 1(d)). Multivariate linear regression analysis showed that after adjusting for age, BMI and TAG the difference between the treatment groups remained significant for ucOC (*P* = 0·001) and dp-ucMGP (*P* < 0·0001), even after correction for multiple comparisons (Table 3).

### Circulating markers of inflammation, endothelial dysfunction and lipid metabolism

Markers of inflammation, endothelial dysfunction and lipid metabolism were not affected after consuming the fortified yogurt drink. Also after adjusting for the potential confounders age, BMI and TAG none of the circulating markers reached the level of significance (Table 3).

## Discussion

In the present paper, we report that yogurt forms an excellent matrix for food fortification with vitamin K since MK-7 was well absorbed and transported to its target tissues, including bone and arteries. This is the first time that a dose of vitamin K lower than the RDA (Commission Directive 2008/100/EC) is shown to be able to significantly improve vitamin K status (as measured by significant decreases in circulating ucOC and dp-ucMGP) in healthy adults. We previously showed a significant improvement of vitamin K status in healthy children after intake of MK-7 supplements (daily dose of 45 µg). This dose however – when taking into account body weight – equals 150 µg for adults. Vascular vitamin K status (as measured by circulating dp-ucMGP) correlated at baseline with markers for inflammation and endothelial dysfunction. During the relatively short intervention period, however, these markers for vascular health were not changed by consumption of the fortified yogurt drink.

Most intervention studies with vitamin K published today have used food supplements rather than fortified foods, and the decreases in ucOC and dp-ucMGP ranged between 20 and 85 % depending on the vitamin K dose^(^^7^^–^^10^^,^^23^^,^^32^^–^^41^^)^. While phylloquinone was supplemented at high doses (0·5–1·0 mg/d), effects of MK-7 intervention were demonstrated at lower intake levels: steady-state levels for plasma MK-7 of 6 ng/ml were shown at 150 µg/d with a significant effect on OC carboxylation^(^^42^^)^. Furthermore, Theuwissen *et al*. showed an increase in circulating MK-7 from 0·4 to 1·5 ng/ml at an intake of 90 µg/d accompanied by significant decreases in ucOC and dp-ucMGP (30 and 34 %, respectively)^(^^7^^)^. At lower intakes (10 to 45 µg/d), no significant effects were observed. Next to supplements, MK-7-enriched yogurt was used to supply participants with an extra 100 µg MK-7/d and after 12 months of treatment the ucOC:cOC ratio decreased by about 50 %^(^^32^^)^. Unfortunately, results on circulating MK-7 were lacking. In our current study, circulating MK-7 had increased from 0·28 to 1·94 ng/ml already after 12 weeks, leading to significant decreases in circulating ucOC and dp-ucMGP (33 and 24 %, respectively). These figures are comparable with those obtained with supplements containing 90 µg MK-7^(^^7^^)^, and demonstrate that a yogurt drink is an excellent matrix for fortification with MK-7. The added fish oil may have facilitated absorption and/or efficacy of the lipophilic MK-7 accounting for the observed circulating MK-7 levels, because it is well known that vitamin K absorption is stimulated by concomitant fat intake and bile secretion^(^^43^^)^. Whether specific components in the fortified yogurt drink, such as the fish oil or the dairy product matrix itself, influenced the bioavailability of MK-7 needs further investigation.

Since low dietary menaquinone intake has been associated with vascular calcification and mortality, we have investigated baseline associations between markers of vitamin K status and vascular health. We used circulating dp-ucMGP and ucOC as markers of vitamin K status of the vasculature and bone, respectively. Significant correlations were found between plasma dp-ucMGP levels and markers for low-grade inflammation (IL-6) and endothelial dysfunction (VCAM and von Willebrand factor). Circulating ucOC did not correlate with markers of vascular health. Observational studies have shown associations between various measures of vitamin K status (as determined by plasma phylloquinone, phylloquinone intake, (%) ucOC, and dp-ucMGP) and inflammatory markers^(^^8^^,^^44^^–^^46^^)^. Remarkably, in our study the other measured MGP form, i.e. dp-cMGP, also correlated with the vascular markers.

Finally, we addressed the question whether the fortified yogurt drink may have beneficial effects on the markers for vascular health. This turned out not to be the case. Both vitamin K and fish oil have been linked to suppression of inflammation, but no clear underlying mechanisms have been found so far. Regarding vitamin K, long-term phylloquinone supplementation at 0·5 mg/d showed no effects on circulating cytokines, despite beneficial effects on vascular health as measured by decreased progression of coronary arterial calcification in elderly. In contrast, 3-month MK-4 administration at 45 mg/d significantly decreased C-reactive protein and matrix metalloproteinase-3 levels in female rheumatoid arthritis (RA) patients^(^^47^^)^. Difference in dosage or the inflammatory state of the participants (healthy *v.* RA) may account for the diverging results. On the other hand, our study may lack sufficient power to detect effects on inflammatory markers since we based our power calculations on changes in vitamin K status. Regarding fish oil, the latest review by Rangel-Huerta *et al*.^(^^48^^)^ concludes that dietary *n*-3 PUFA are associated with lower levels of inflammatory markers in CVD and other chronic diseases. Fish oil supplementation had generally no effect on inflammatory markers in the studies of healthy participants. Fish oil supplementation was found to have only isolated effects on plasma intercellular adhesion molecule (*P* = 0·05; 2·1 g/d for 8 weeks)^(^^49^^)^ and VCAM levels (*P* < 0·05; 1 g/d for 12 weeks)^(^^50^^)^. The authors indicate – as mentioned for vitamin K – that the lack of an anti-inflammatory effect may be due to the lower serum levels in healthy subjects, minimising the possibility of their reduction. Compared with the dosages described (range 0·4–23·6 g/d; doses >5 g/d through infusion), we used a relatively low daily dose of *n*-3 PUFA which may also contribute to the null findings.

Our study has some limitations. First, no results were available on the background diet of the participants. We collected information on their intake of vitamin K-containing food products, but not on their general dietary habits. Second, our study population was limited to healthy elderly, which makes extrapolation to other population groups difficult. Finally, the fact that besides MK-7 other nutrients were added to the fortified yogurt means that it remains unclear whether the effect of the intervention on vitamin K status would have been different if only MK-7 had been added.

### Conclusion

We have demonstrated that consuming a yogurt drink fortified with low doses of among others vitamin K (MK-7, daily dose of 56 µg), vitamins C and D, and *n*-3 PUFA (EPA + DHA, daily dose of 0·2 g) significantly improves vitamin K status. Inflammatory biomarkers and lipid markers had, however, not changed after drinking the yogurt for 12 weeks. The importance of *n*-3 PUFA for menaquinone absorption needs further investigation.
